# DMN network and neurocognitive changes associated with dissociative symptoms in major depressive disorder: a research protocol

**DOI:** 10.3389/fpsyt.2025.1516920

**Published:** 2025-04-01

**Authors:** Asli Ercan Dogan, Herdem Aslan Genc, Sinem Balaç, Sevin Hun Senol, Görkem Ayas, Zafer Dogan, Emre Bora, Deniz Ceylan, Vedat Şar

**Affiliations:** ^1^ Department of Psychiatry, School of Medicine, Koç University, Istanbul, Türkiye; ^2^ Department of Child and Adolescent Psychiatry, School of Medicine, Koç University, Istanbul, Türkiye; ^3^ Graduate School of Health Sciences, Koç University, Istanbul, Türkiye; ^4^ Koç University Research Center for Translational Medicine (KUTTAM), Affective Laboratory, Istanbul, Türkiye; ^5^ Department of EEE, MLIP Research Group & KUIS AI Center, Koç, University, Istanbul, Türkiye; ^6^ Department of Neurosciences, Institute of Health Sciences, Dokuz Eylül University, Izmir, Türkiye; ^7^ Department of Psychiatry, School of Medicine, Dokuz Eylül University, Izmir, Türkiye; ^8^ Department of Psychiatry and Psychology, Mayo Clinic, Rochester, MN, United States

**Keywords:** depression, dissociation, childhood trauma, functional connectivity, neurocognitive function

## Abstract

**Introduction:**

Depression is a heterogeneous disorder with diverse clinical presentations and etiological underpinnings, necessitating the identification of distinct subtypes to enhance targeted interventions. Dissociative symptoms, commonly observed in major depressive disorder (MDD) and linked to early life trauma, may represent a unique clinical dimension associated with specific neurocognitive deficits. Although emerging research has begun to explore the role of dissociation in depression, most studies have provided only descriptive analyses, leaving the mechanistic interplay between these phenomena underexplored. The primary objective of this study is to determine whether MDD patients with prominent dissociative symptoms differ from those without such symptoms in clinical presentation, neurocognitive performance, and markers of functional connectivity. This investigation will be the first to integrate comprehensive clinical evaluations, advanced neurocognitive testing, and high-resolution brain imaging to delineate the contribution of dissociative symptoms in MDD.

**Methods:**

We will recruit fifty participants for each of three groups: (1) depressive patients with dissociative symptoms, (2) depressive patients without dissociative symptoms, and (3) healthy controls. Diagnostic assessments will be performed using the Structured Clinical Interview for DSM-5 (SCID) alongside standardized scales for depression severity, dissociation, and childhood trauma. Neurocognitive performance will be evaluated through a battery of tests assessing memory, attention, executive function, and processing speed. Structural and functional magnetic resonance imaging (MRI) will be conducted on a 3 Tesla scanner, focusing on the connectivity of the Default Mode Network with key regions such as the orbitofrontal cortex, insula, and posterior cingulate cortex. Data analyses will employ SPM-12 and Matlab-based CONN and PRONTO tools, with multiclass Gaussian process classification applied to differentiate the three groups based on clinical, cognitive, and imaging data.

**Discussion:**

The results of this study will introduce a novel perspective on understanding the connection between major depressive disorder and dissociation. It could also aid in pinpointing a distinct form of depression associated with dissociative symptoms and early childhood stressors.

**Conclusion:**

Future research, aiming to forecast the response to biological and psychological interventions for depression, anticipates this subtype and provides insights.

## Introduction

Major Depressive Disorder (MDD) has a significant impact on both disability and mortality among young people (1–3). The onset of psychiatric disorders often occurs in adolescence and early adulthood, with three-quarters of adults experiencing their first symptoms before age 24. The average age of onset is 14.5 years for any psychiatric disorder and 19.5 years for MDD (1, 2). MDD affects about 16% of individuals aged 16–24. In this group, depression not only reduces quality of life and functional capacity but also significantly contributes to early mortality, with suicide being the third leading cause of death among individuals aged 15–29 (4). These alarming statistics emphasize the urgent need for a better understanding of MDD’s causes and effects in young people.

Despite extensive research into the causes of Major Depressive Disorder (MDD), understanding its underlying mechanisms remains a significant challenge. This limited comprehension contributes to clinical difficulties, such as low treatment response rates and obstacles in developing new therapeutic targets, thereby adding to the global disease burden (5). The clinical and biological diversity of MDD, coupled with the limitations of traditional subtypes (e.g., endogenous/reactive, typical/atypical), fails to capture the disorder’s full complexity (6). Depression, though categorized under a single diagnosis, manifests with varied symptoms, severity levels, and comorbidities, making standardized treatment difficult (7). To address this heterogeneity, researchers emphasize the need to identify hidden subtypes using new variables and transdiagnostic approaches which could enhance understanding and lead to more targeted treatments (8).

### Dissociation: a critical concern in young age

Dissociative symptoms such as depersonalization, derealization, confusion, flashbacks, and amnesia are significantly more common in individuals with MDD compared to the general population, particularly in young populations (9–14). Approximately 45% of adolescents in clinical settings meet the diagnostic criteria for a specific dissociative disorder as outlined in the DSM-5 (13, 14) and over 60% of patients with depressive disorders exhibit clinically significant dissociation (15). Involving a disruption in memory, identity, consciousness, or perception of the environment, dissociation often serves as a coping mechanism for trauma and emotional distress (16, 17). Notably, more than half of individuals with MDD report traumatic experiences in childhood (18). Recent research yield that those with both depression and dissociation report even higher childhood trauma scores compared to those with only one of them (19). These findings suggest that those with both MDD and dissociative symptoms may constitute a distinct subgroup with unique clinical needs. In fact, underdiagnosed dissociative symptoms can act as a confounding factor in entire spectrum psychiatry (10), complicating the interpretation of findings, including MDD.

As a treating point to address this problem, a dissociative subtype has been formalized for Post-Traumatic Stress Disorder (PTSD) in DSM-5 already (20). Proposals for a dissociative subtype have been made for schizophrenia as well (21, 22). Following this line of thought, Sar (23) formulated the concept of “dissociative depression”, defined as the presence of a chronic dissociative disorder in individuals who meet the criteria for a MDD diagnosis. The prevalence of dissociative depression fitting this definition was found to be 4.1% in a sample of women representing the entire city of Sivas, Turkey (12). This potential subtype of MDD is associated with early onset, treatment resistance, and more severe symptoms, including impulsivity, rapid mood changes, psychotic symptoms, self-harm, suicidality, higher rates of antipsychotic prescriptions, and higher comorbidity with PTSD, borderline and antisocial personality disorders (24–28). As a potential indicator of suicidality, self-harm behavior, and need for psychosocial intervention in young adults, this concept warrants further investigation (9, 15, 29). However, more research is needed to establish clinical parameters for distinguishing between MDD with and without dissociative symptoms. Given that the adolescence is the age group with highest prevalences of dissociative disorders in clinical settings, and that these disorders often have an early onset, including childhood, dissociative depression becomes also a critical concept for age groups before adulthood (13, 30).

### Neuropsychological findings: memory disturbances as the target symptom

Cognitive dysfunction is prevalent in various mental disorders, including MDD and dissociative disorders, affecting verbal memory, attention, and executive function (31). In MDD, approximately 27% of patients exhibit global neurocognitive deficits (32), including impairments in span attention, learning and memory, processing speed, psychomotor speed, and executive functions, often persisting even after symptomatic remission (33–36). In dissociative disorders, memory impairments are a prominent feature, with higher dissociation levels linked to deficits in verbal memory, delayed recall, general memory, and long-term memory (37, 38). Pathological dissociative experiences, such as amnesia and depersonalization/derealization, are inversely related to overall memory performance; individuals with high dissociation levels perform worse on immediate visual memory tasks compared to those with low dissociation levels (39).

A systematic review and meta-regression analysis revealed that depression scores, rather than dissociative experiences, are significantly associated with decreased memory specificity (40). However, dissociation is also linked to impairments in attention and executive functioning, with highly dissociative individuals exhibiting heightened distractibility and reduced cognitive inhibition (41). Studies have shown that dissociative symptoms are associated with poor performance on attentional tasks, especially when trauma-relevant distractors are present (42, 43). Deficits in executive functioning, such as impaired cognitive flexibility and problem-solving abilities, have also been observed in this population (44). Adolescents with a dissociative disorder have shown impairments in working memory, sustained attention, visual learning and memory, and verbal memory (45). Another study found that adolescents with dissociative disorder performed worse on executive function tasks (Wisconsin card sorting test), arithmetic, coding, and maze tests compared to healthy adolescents (31).

The relationship between dissociation and cognitive dysfunction is complex and not always straightforward. Some studies have found that higher dissociation levels correlate with reduced attention and verbal memory performance (46). However, other research has not observed a significant association between dissociation severity and neurocognitive test outcomes (47). A recent systematic review highlighted that self-reported cognitive difficulties align with dissociative experiences, while objective cognitive task results remain inconsistent (48). Despite the evidence linking dissociation to cognitive impairments, further research is necessary to clarify the mechanisms driving these dysfunctions and to explore how they manifest across different populations and contexts. A single cross-sectional study investigating the relationship between dissociative symptoms and cognitive functions in individuals with MDD found that derealization was associated with impairments in verbal and visual memory, whereas depersonalization was linked to reduced processing speed (49). Interestingly, depersonalization symptoms correlated with enhanced attentional performance in low-stimulus environments. However, the study’s small sample size—23 patients with MDD and 20 healthy controls—limits the generalizability of these findings. Notably, the role of dissociative symptoms in cognitive dysfunction within MDD remains underexplored, presenting an unresolved clinical question.

### Resting state DMN functional connectivity: MDD and dissociation

The Default Mode Network (DMN) is a large-scale brain network primarily composed of the medial prefrontal cortex (mPFC), posterior cingulate cortex (PCC), and precuneus. This network is most active during rest and is associated with self-referential thinking, daydreaming, memory recall, and spontaneous processing of stimuli. Conversely, its activity diminishes during tasks requiring external attention. The DMN supports advanced cognitive functions such as introspection, autobiographical memory, decision-making, and perception of the external world. Functional magnetic resonance imaging (fMRI) has significantly advanced our understanding of the DMN and other networks, like the salience and dorsal attention networks, by measuring changes in the blood-oxygen-level-dependent (BOLD) signal (50).

MDD is associated with significant alterations in the functional connectivity of the DMN. These alterations have been detected both within the subregions of the DMN and between the DMN and other key emotion-regulating networks such as the salience and affective networks. Aberrant connectivity within the DMN, particularly hyperconnectivity in the mPFC and PCC, is frequently reported in MDD. This hyperconnectivity has been implicated in the maladaptive self-referential processing and rumination commonly observed in depression. The connection between the anterior and posterior nodes of the DMN has consistently shown alterations in MDD. Studies using the anterior DMN as the seed region report dissociation between the anterior and posterior DMN, while those using the posterior DMN as the seed report increased connectivity between the two nodes. In healthy volunteers, a distinct anterior-posterior subnetwork within the DMN contributes to different aspects of self-generated thought (51, 52). Leech and Sharp (53) hypothesized that increased PCC connectivity with anterior DMN regions relates to internally directed attention and rumination in depression, although the effects of functional connectivity changes between anterior and posterior subnetworks remain poorly understood (54). Reduced connectivity between anterior and posterior DMN nodes in MDD is supported by structural connectivity reductions in an sgACC-posterior DMN-based network (55).

**Figure 1:**
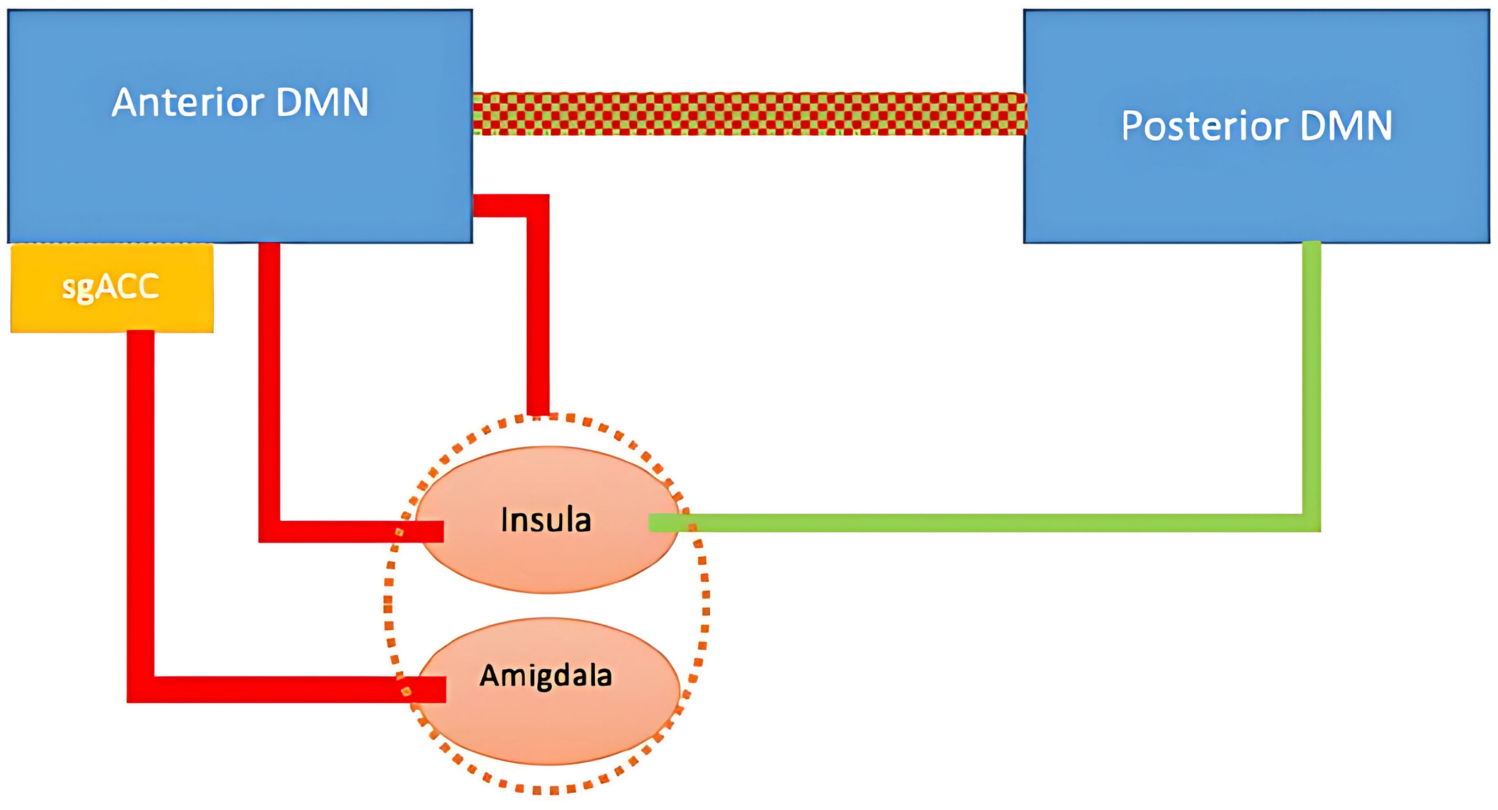
DMN and associated regions in depression. Red lines represent increase in connectivity; green line represent a decrease in connectivity. Red and green dotted line represents altered connectivity.

**Figure 1** summarizes findings related to functional connectivity of the DMN in MDD. A meta-analysis identified increased connectivity between the DMN and regions such as the medial prefrontal cortex (mPFC), dorsolateral prefrontal cortex (DLPFC), and hippocampus (56). This finding is further supported by a systematic review demonstrating heightened connectivity within the anterior DMN (57). Despite the typical reduction in gray matter volume in these regions, functional activity within the DMN paradoxically increases during resting states in individuals with MDD (58–61). Similar connectivity patterns have been observed in adolescents with MDD, suggesting that these alterations may emerge early in the disorder’s course (62).

However, connectivity differences may depend on illness chronicity. A study involving 1.300 participants found increased DMN connectivity in individuals with recurrent MDD but not in those experiencing a first-episode, drug-naïve depression (63). Furthermore, research has identified two distinct neurobiological subtypes of depression based on DMN connectivity patterns, which may help explain the heterogeneity of clinical presentations. The predominant subtype, observed in 70–80% of MDD patients, is characterized by hyperconnectivity within the DMN, particularly among its core hubs, and is considered the more “typical” form of depression. In contrast, a smaller subgroup (20–30% of patients) exhibits DMN hypoconnectivity, which has been linked to higher rates of comorbid anxiety disorders, recurrent or chronic depressive episodes, and a greater prevalence among female patients (63). Similarly, a review by Dichter et al. (64) examined predictors of treatment response in MDD using resting-state fMRI. The review concluded that increased functional connectivity between frontal and limbic brain regions was associated with a positive response to antidepressant treatment. Conversely, hyperconnectivity within the DMN was linked to treatment resistance to transcranial magnetic stimulation (TMS) in MDD. These findings suggest that alterations in DMN connectivity not only contribute to the pathophysiology of depression but also have implications for predicting and personalizing treatment strategies. Understanding these connectivity patterns can aid in developing targeted interventions for individuals with MDD.

### Resting state DMN functional connectivity: within and between networks

Dissociation is a psychological condition characterized by the presence of unresolved internal processes related to trauma, such as repetitive and unproductive thinking patterns known as “rumination”. As mentioned above, DMN is active during self-referential thinking and becomes deactivated during external processes that need attention (65); therefore, heightened activity in the DMN challenges of shifting from internal ideas, often experienced during dissociation, to outward thoughts becomes more apparent. Hyperactivity in the bilateral superior frontal regions and the medial segments of the inferior frontal and middle frontal regions were identified as neurofunctional biomarkers of pathological dissociation (66). A transdiagnostic study investigated the brain connectivity markers of dissociation during resting state and reported that functional connectivity between the orbitofrontal locus and retrosplenial cortex was negatively related to the DES score, whereas connectivity between the orbitofrontal region and other default mode regions was positively related to the DES score (67). Another study conducted on women with borderline personality disorder revealed a link between DES scores and the resting-state functional connectivity of the amygdala with the dorsolateral prefrontal cortex and fusiform gyrus (68). Paul et al. (69) discovered a correlation between higher symptoms of depersonalization and reduced connectivity between the extrastriate body area (a brain region linked with body parts and motions) and the DMN in individuals with MDD.

Previous studies have revealed the significance of the orbitofrontal cortex (OFC) in dissociative disorders, as indicated by our team’s findings. Two studies by Sar et al. (11, 70 looked at brain blood flow and found that people with chronic dissociative disorder had less activity on both sides of the orbitofrontal cortex compared to a healthy control group. The “orbitofrontal hypothesis” posits a potential link between dissociative depression and the OFC, a region known for its crucial role in affect regulation and its high susceptibility to early-life stressors (71, 72). This hypothesis emphasizes the need to study the networks associated with the OFC in dissociative disorders. The dysfunctional connectivity of cortical-subcortical circuitries in OFC due to chronic stress during developmental periods forms an enduring vulnerability for psychiatric disorders (72).

The insula is thought to have a key role in processing emotional states, acting as a bridge between subcortical brain regions that receive visceral sensations and frontal lobe regions that determine the emotional and motivational significance of these sensations. It is believed that the insula plays a role in regulating two resting state networks: the anterior insula, which affects brain regions in both the default mode network (involved in internal observation) and the central executive network (involved in emotional evaluation), and the posterior insula, which maintains connections with sensorimotor areas involved in environmental monitoring. Forner (73) conducted a thorough review that explores the inverse connection between mindfulness and dissociation, highlighting the significance of reduced connectivity between the medial frontal cortex and insula in dissociation. To our knowledge, there are no studies yet which specifically investigate the resting state functional connectivity in individuals with MDD and accompanying dissociative symptoms. However, examining research on DMN connectivity in relation to dissociation can provide insights (74, 75). Similar studies on MDD suggest that altered connectivity, especially in treatment-resistant cases, might be linked to concurrent dissociative symptoms.

### Machine learning classification in detecting subtypes

Machine learning algorithms are being utilized to classify psychiatric subgroups and predict treatment responses using behavioral, genetic, electrophysiological, and imaging-based data. In depression research, most studies focus on differentiating depressed individuals from healthy controls, while some aim to predict treatment response. However, fewer studies specifically target the distinction between depression subtypes, highlighting a gap in research (76). A data-driven study analyzing DMN patterns in depression identified two biological subtypes with increased and decreased DMN activation (77). Machine learning has been widely applied in psychiatry for predicting suicidality (78), bipolar disorder (79, 80), psychotic symptoms (81), and prediction of postpartum depression (82), with its their use steadily increasing. However, the differentiation of dissociative symptoms in depression using machine learning remains unexplored. In PTSD, machine learning has shown promising results: a multiclass Gaussian process classification model distinguished dissociative subtype of PTSD with up to 91.63% accuracy based on spontaneous neural activity, and 85% accuracy based on amygdala connectivity (83). Another study using the same method has distinguished individuals with and without dissociative subtype of PTSD from healthy individuals with 80.4% accuracy based on insula connectivity (84).

## Objectives and hypothesis

Depression is a leading cause of morbidity and mortality in young adults, yet its heterogeneous nature complicates understanding its pathophysiology and treatment resistance. Notably, dissociative symptoms are prevalent in youth depression, shaping distinct clinical and prognostic trajectories. Subtyping youth depression based on the presence of dissociative symptoms is crucial for enhancing the consistency of future research and the development of personalized treatments. In light of these considerations, this study addresses three critical questions. First, is MDD with dissociative symptoms in young adults a distinct clinical subtype characterized by an earlier onset of depression, more severe symptomatology, and higher rates of self-harm and suicidal ideation? Second, are dissociative symptoms linked to greater cognitive deficits—particularly in memory, attention, and executive functions? Finally, can differences in resting-state DMN connectivity, especially in the mPFC and PCC, serve as neurobiological markers to distinguish between these subgroups?

To answer these questions, the study will compare young adults with MDD who exhibit prominent dissociative symptoms (Dis+) against those without dissociative features (Dis–), as well as a group of healthy controls. We will employ a multimodal approach that integrates comprehensive clinical evaluations, neuropsychological testing, and advanced neuroimaging techniques. In particular, resting-state functional magnetic resonance imaging (fMRI) will be used to assess DMN connectivity, while machine learning algorithms will facilitate the classification of participants based on clinical, cognitive, and imaging data.

By delineating the clinical and neurobiological distinctions between MDD subtypes, this work promises to refine the understanding of depression’s heterogeneity and pave the way for more targeted, personalized treatment interventions. In doing so, it may not only improve diagnostic precision but also enhance therapeutic outcomes for young patients grappling with the dual challenges of depression and dissociation.

The study will address the following four hypotheses: (1) patients in the Dis+ group will exhibit an earlier onset of depression, more frequent depressive episodes, increased psychiatric comorbidity, higher rates of self-harm behavior and suicidal ideation, more frequent childhood trauma, insecure interpersonal attachment patterns, and greater difficulty in emotion regulation compared to the Dis- group; (2) both depression groups (Dis+ and Dis-) are expected to show impaired performance on neuropsychological tests compared to healthy controls and within the depression subgroups, the Dis+ group will exhibit more pronounced impairments, particularly in verbal and visual memory, when compared to the Dis- group; (3) both depressed groups will show reductions in cortical thickness, surface area, and gray matter volume in specific brain regions compared to healthy controls and additionally, both groups will show increased resting-state connectivity within the default mode network (DMN), with differing levels of functional connectivity between the orbitofrontal cortex (OFC), insula, posterior cingulate cortex (PCC), and DMN; (4) a machine learning model based on neurocognitive test results and imaging data will differentiate between the Dis+, Dis-, and healthy control groups with at least 80% accuracy.

This study will be the first to investigate functional connectivity changes associated with dissociative symptoms in MDD. Thus, this investigation is timely and significant. Through this comprehensive approach, the study aims to contribute to a paradigm shift in the diagnosis and treatment of depression, particularly in the context of its complex interplay with dissociative phenomena. This refined perspective is expected to inform both clinical practice and future research, ultimately enhancing the quality of care for young individuals with MDD.

## Material and methods

This is a single-centered study that will be carried out at the Psychiatry Department or KUPTEM outpatient clinics of Koç University School of Medicine. Inclusion and exclusion criteria will be established to identify eligible individuals for participation in the study. The project has received approval and is under the supervision of the Human Ethics Committee of Koç University and Koç University Hospital Medical Advisory Committee. Prior to participation, written informed consent will be obtained from the participants and/or their legal guardians. This study is also funded by the Scientific and Technological Research Council of Turkey (TÜBİTAK) with 1001 - The Scientific and Technological Research Projects Funding Program and Koç University.

Sample size calculation was performed by using OpenEpi, based on the average and standard deviations of PHQ-9 scores from the study by Fung et al. (15). The calculation determined that a minimum of 31 participants per group is required to detect significant differences in psychopathology between the Dis+ and Dis- groups, with a 95% confidence interval and 80% power. For machine learning analyses, an area under the ROC curve (AUC) calculation indicated that each group requires at least 24 participants to achieve 95% confidence and 80% power, assuming an AUC of 0.75 or higher. To enhance statistical power and account for data losses or the need for adjustments in multiple comparisons, each group will consist of 50 participants.

### Study design

**Figure 2:**
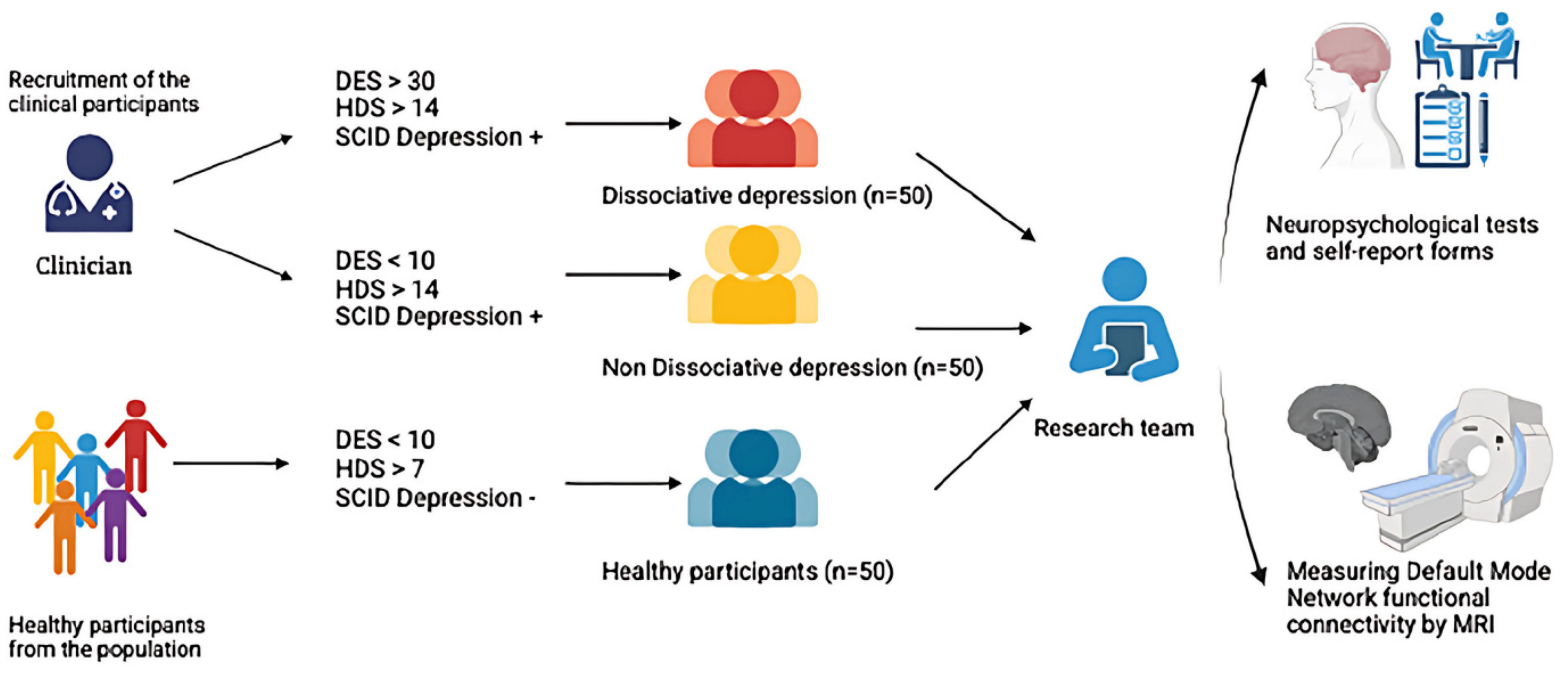
Recruiting participants and conducting clinical and diagnostic interviews. DES, Dissociative Experiences Scale; HDS, Hamilton Depression Scale; SCID, Structured Clinical Interview for DSM Disorders. From: BioRender.com.

This research is a cross-sectional study comparing three groups: (1) patients with MDD and dissociative symptoms (Dis+), (2) patients with MDD without dissociative symptoms (Dis-), and (3) healthy control participants. The study will include clinical assessments, neurocognitive testing, and resting-state functional connectivity analysis using MRI. **Figure 2** summarizes the recruitment and study procedures.

Patients aged 15 to 25, of both biological sexes, diagnosed with Major Depressive Disorder (MDD) based on DSM-5 criteria and seeking treatment at the Psychiatry Department or KUPTEM outpatient clinics of Koç University School of Medicine, will be recruited for this study. Healthy controls of the same age range and similar gender distribution will also be included.

The study will consist of three groups:

1. Dis+ Depression Group: 50 participants diagnosed with MDD who also exhibit dissociative symptoms.
2. Dis- Depression Group: 50 participants diagnosed with MDD but without dissociative symptoms.
3. Healthy Control Group: 50 participants without any psychiatric or mental health conditions.

### Inclusion and exclusion criteria for participation

The inclusion criteria of Dis+ (patients with MDD with dissociation) as follows: (1) aged 15–25, (2) right-handed, (3) diagnosed with MDD via SCID-5, (4) Hamilton Depression Scale score ≥ 14, (5) Dissociative Experiences Scale (DES) score ≥ 30, (6) SCID-D score of at least 2 (mild) on at least one dissociative dimension (amnesia, depersonalization, derealization, identity confusion, or identity alteration).

For the Dis- Depression Group (MDD without dissociative symptoms) are as follows: (1) aged 15–25, (2) right-handed, (3) diagnosed with MDD via SCID-5, (4) Hamilton Depression Scale score ≥ 14, (5) DES score < 10, (5) SCID-D dissociative dimension scores all below 2 (mild).

The inclusion criteria for healthy control group are as follows: (1) aged 15–25, (2) right-handed, (3) no psychiatric diagnosis, (4) no scores ≥ 2 on any SCID-D dissociative dimension, (5) Hamilton Depression Scale score ≤ 7, (6) DES score < 10, and (7) no psychiatric history or significant family history of MDD, bipolar disorder, psychotic disorders, or neurodevelopmental disorders.

The exclusion criteria of patients for all the groups are (1) visual or hearing impairments, (2) left-handedness, (3) use of benzodiazepines or psychostimulants within 72 hours prior to imaging or neuropsychological assessments, (4) diagnosis of bipolar disorder, schizophrenia, or other psychotic spectrum disorders, (5) neurological disorders or decompensated systemic medical conditions, (6) history of neurosurgical intervention or head trauma with loss of consciousness, (7) active alcohol or substance use disorder within six weeks prior to imaging, (8) contraindications to MRI, (9) pregnancy, postpartum, or breastfeeding.

### Assessment tools

#### Semi-structured interview schedules and symptom lists administered by clinicians

To ensure a robust and standardized diagnostic evaluation, we will employ the Structured Clinical Interview for DSM (SCID) to diagnose Major Depressive Disorder (MDD) and identify psychiatric comorbidities. For our adolescent population, a child and adolescent psychiatrist will administer the KSADS-5—the Turkish adaptation of the SCID—ensuring that developmental and cultural considerations are appropriately addressed. We will use the Hamilton Depression Scale to assess the severity of depression. This project will develop a sociodemographic and psychiatric history form that will compile sociodemographic and general medical details, including the age of onset of psychiatric symptoms, treatments received, number of depressive episodes, and childhood experiences such as parental loss or placement in alternative care. Given our study’s focus on dissociative phenomena, we will use the Dissociative Experiences Scale (DES) to assess chronic dissociation; scores of 30 or higher will be indicative of pathological dissociation. Complementarily, the Somatoform Dissociation Questionnaire (SDQ) will be administered to evaluate somatic symptoms arising from dissociation (e.g., conversion symptoms and medically unexplained pain). To better understand the influence of interpersonal dynamics on clinical presentation, we will assess attachment styles using the Relationship Scales Questionnaire (RSQ-30). Emotional regulation will be evaluated using both the Difficulty in Emotion Regulation Scale Short Form (DERS-16) and the Hypomania Symptom Checklist (HCL), the latter helping to identify mood elevation or subthreshold manic features that may complicate the clinical picture. Finally, the Childhood Trauma Questionnaire Revised Form (CTQ-33) will be employed to quantify early adverse experiences, which are known to contribute to both depressive and dissociative symptomatology.

#### Application of neuropsychological tests to participants

Following the diagnostic and clinical interviews, participants will undergo a comprehensive battery of neuropsychological tests administered within the same week. This battery, which lasts approximately two hours, is designed to assess multiple cognitive domains implicated in MDD and dissociative disorders. The testing will be conducted by trained research assistants under the supervision of an experienced researcher, within a controlled environment between 9:00 a.m. and 12:00 p.m., with a 30-minute break provided to ensure participants are rested and not affected by fatigue or hunger. The selected tests include:

Rey Auditory Verbal Learning Test (RAVLT): Assesses verbal learning and memory, providing measures of both immediate and delayed recall.
Wisconsin Card Sorting Test (WCST): Evaluates executive functions, particularly cognitive flexibility and problem-solving abilities.
Digit Span Test: Measures attention and working memory capacity.
Stroop Test: Assesses cognitive inhibition and attentional control by requiring participants to suppress automatic responses.
Trail Making Tests A and B: Evaluate processing speed, visual attention, and task-switching capabilities.
Category Fluency Test: Assesses semantic memory and executive function through the rapid generation of words within specific categories.
Symbol Digit Modalities Test (SDMT): Measures processing speed and attention via rapid symbol-digit pairing.
Verbal Fluency Test: Evaluates language production and executive control during word retrieval tasks.
Auditory Consonant Trigram Test: Assesses auditory working memory and sustained attention.

These tests were selected because they have demonstrated sensitivity to the cognitive deficits commonly observed in MDD and dissociative disorders, and their validity has been well-established in prior clinical research (85). By using this comprehensive battery, we aim to capture a broad profile of neurocognitive functioning, which will enable us to examine the associations between cognitive performance, dissociative symptoms, and the neurobiological markers of MDD.

### Statistical analysis

SPSS and GraphPad will be used to describe clinical data with counts/percentages for categorical data and means/medians for continuous data. Neuropsychological test scores will be recorded with means, standard deviations, and interquartile ranges, with Z scores for visualizations. Group comparisons will use ANOVA or ANCOVA for normally distributed data, with covariates used to control for confounding factors. Non-normally distributed data will be transformed prior to analysis. Multivariate methods and principal component analysis will be used, with adjustments for multiple comparisons. In order to control for the increased risk of Type I errors due to multiple comparisons, a Bonferroni correction was applied to all correlation analyses, with the significance threshold adjusted accordingly (α_corrected = 0.05/n).

#### Acquisition of functional and anatomical images and preprocessing

A 3 Tesla Siemens Skyra device with 64-channel and 20-channel head coils will be used for functional and anatomical imaging. Participants are instructed to remain still and awake during the scan. Spatial image preprocessing, including BOLD and MPRAGE images, will be done using SPM12. This includes realignment, co-registration, normalization, and spatial smoothing. Functional images will be registered to the T1-weighted template, segmented, normalized to an MNI template, and smoothed with a 6 mm Gaussian kernel. Motion regressors will be created using ART software. The data will be filtered to minimize low-frequency drift and high-frequency noise. Functional images will be realigned and resliced, excluding the first four volumes, to prevent saturation effects. Data will be excluded for excessive head motion, and mean displacement will be included as a covariate in further analyses, tested for group differences using ANOVA.

#### Examination of fMRG brain volumes, surface area, and cortical thickness

We will use voxel-based analysis with the FSL program for regional gray matter analysis. Pre-analysis steps include brain tissue separation, tissue segmentation, registration, modulation, and smoothing. Images will be inspected for misalignments and corrected manually if needed. Processed brain structures will be partitioned into gyri using a cortical parcellation tool based on the Destrieux atlas. Cortical thickness will be examined with FreeSurfer, including motion correction, non-brain tissue removal, tissue segmentation, and topographic surface calculation. Quality control will follow the ENIGMA protocol and visual inspection. Preprocessing steps involve removing non-brain structures, volume labeling, intensity normalization, white matter segmentation, surface atlas registration, and gyri labeling. Group comparisons will use a general linear model with a p-value < 0.05, adjusted for multiple comparisons.

#### Examination and statistical analysis of fMRI functional connectivity

This study will use seed-based correlation analysis (SCA) to examine resting-state connectivity. Seed regions will be selected based on anatomical areas associated with pathology, such as the OFC, insula, and posterior cingulate cortex (PCC). Regions of interest (ROIs) will be identified using the Automated Anatomical Labeling (AAL) atlas. Functional connectivity will be analyzed using the MATLAB-based CONN toolbox, generating seed-voxel and ROI-ROI maps. Correlation matrices will be created by converting R-values to standard z-values. Seed-based connectivity maps will be generated for each participant, followed by group-level analyses. A voxel threshold of p ≤ 0.001 and a cluster size threshold of p ≤ 0.05 will be applied. Group comparisons (controls, dis-depression, and dis+ depression) will be conducted using ANOVA, with corrections for multiple comparisons.

#### Multiclass Gaussian Process Classification Machine Learning (MGPC)

Functional and structural brain images, along with neuropsychological tests, will be used to differentiate between healthy controls, dis-depression, and dis+ depression groups through multivariate machine learning classification using the PRONTO toolbox within SPM12. PRONTO includes four classification algorithms: SVM, BGPC, MGPC, and L1-multiple kernel learning. For our three-group classification, MGPC will be the primary model. If MGPC proves insufficient, we will use BGPC for binary classifications, coding the ‘healthier’ condition as y = -1. Our analysis will follow five steps: determining the dataset, selecting the feature set, evaluating the model with LOOCV, estimating the model, and preparing weight calculations and AUC scores.

#### Dataset determination and features selection

Resting-state connectivity maps and neuropsychological test scores will be used as inputs, applying the DARTEL gray matter method. Features will include voxel-level data, age, education, and test scores. Top features will be selected based on Kendall tau correlations and Gaussian process covariance functions. The data will be normalized using linear and principal component methods. MGPC, Simple-MKL, SVM, and BGPC algorithms will be used with LOOCV for model evaluation. Performance will be measured by accuracy, specificity, sensitivity, predicted probabilities, and AUC scores. Permutation testing (1,000 times) will identify significant features, creating a discriminative map based on p-values.

## Discussion

This proposed study aims to investigate whether MDD with dissociative symptoms represents a qualitatively distinct subtype from MDD without dissociative features in young adults. This distinction could significantly enhance our understanding of the role that psychosocial stress during developmental years plays in the onset of depressive disorders. By combining detailed clinical and cognitive assessments with advanced neuroimaging analysis of DMN connectivity using deep learning, we hope to elucidate key differences between these potential MDD subtypes. If our hypothesis is supported by the results, it could lead to a paradigm shift in our approach to the diagnosis and treatment of MDD. First of all, it could suggest the need for specialized interventions targeting dissociative symptoms in this subgroup. Furthermore, DMN connectivity patterns identified through deep learning could potentially serve as a biomarker to aid in distinguishing these subtypes.

Additionally, this research could pave the way for future studies exploring the underlying mechanisms linking dissociation and depression, potentially uncovering novel biomarkers that could inform more effective therapeutic strategies. Dissociative symptoms also can influence the results of clinical and biological research on these disorders as a “confounding” factor, raising questions about possible dissociative subtypes in populations such as PTSD (20), schizophrenia (21, 22), and MDD (24, 25, 27) to achieve more precise study outcomes related to their clinical course and underlying biology. Moreover, by integrating neuroimaging techniques with clinical assessments, we may gain deeper insights into the brain’s functional architecture in individuals experiencing these co-occurring conditions. If our hypothesis regarding differences in neurocognitive performance between Dis+ and Dis- groups is confirmed, it could suggest that dissociative symptoms in MDD are associated with distinct cognitive profiles, potentially indicating different underlying neural mechanisms. This could ultimately lead to tailored interventions that address the specific needs of patients based on their cognitive and dissociative characteristics, enhancing the precision of mental health care. Additionally, exploring the interplay between these cognitive profiles and treatment responses may reveal critical pathways for optimizing therapeutic outcomes, paving the way for personalized approaches in managing both depression and dissociation.

Dissociative experiences in MDD have been associated with increased illness severity, suicidality, and worse treatment outcomes, but it remains unclear whether MDD with dissociative features represents a more severe form of depression or a qualitatively distinct subtype with unique underlying neurobiology. Our proposed multimodal approach combining clinical, cognitive, and neuroimaging data offers several advantages to address this question. The comprehensive clinical and cognitive assessment battery will allow us to characterize the phenomenology and neurocognitive profile associated with dissociative symptoms in MDD. Using measures of hypomania, emotion dysregulation, and anger in addition to depression and dissociation symptoms can provide important information for assessing and prioritizing clinical risks between the two groups.

The inclusion of adolescents is another strength of the study because it has been shown that early-onset depression has negative impacts on overall well-being, academics, and professional life. Suicide, on the other hand, ranks fourth among causes of death in the 15-29 age group (4). These findings demonstrate the need for a new field in psychiatry focusing on young adults. Considering the developmental causes and continuity of depression, limiting young adulthood to the age of 18 is an artificial distinction, and including adolescence in this process would better meet the requirements of this study.

The identification of subtypes that categorically match the etiology could lead to a better understanding of the pathogenesis of depression and allow for the diversification of treatment research. If we identify significant effects of dissociative symptoms in depression, this approach could harmonize with the results of similar studies on other disorders, such as post-traumatic stress disorder and schizophrenic spectrum disorder, stimulate the development of new studies on autism spectrum disorder and attention deficit hyperactivity disorder, and contribute to the exploration of transdiagnostic dimensions in psychiatry and a more precise understanding of the epigenetic effects of stress.

However, several limitations should be considered. The cross-sectional nature of this study limits causal inferences about the relationship between dissociation and depression. Longitudinal research will be needed to determine whether dissociative symptoms precede depression onset or emerge as a consequence. Additionally, while our focus on young adults reduces age-related confounds, it may limit generalizability to other age groups.

In conclusion, this study represents an important step toward understanding the role of dissociative symptoms in MDD and potentially identifying neurobiologically distinct subtypes. The findings could inform more personalized approaches to the diagnosis and treatment of depression, particularly in young adults experiencing dissociative phenomena. Future research building on these results may lead to improved outcomes for this challenging subgroup of MDD patients.
